# Editorial: Reviews in: regulatory science 2024-2025

**DOI:** 10.3389/fmed.2025.1705977

**Published:** 2025-10-20

**Authors:** Mette Due Theilade Thomsen, Lisbeth Ehlert Knudsen

**Affiliations:** ^1^Pip Adviser ApS ltd, Lyngby, Denmark; ^2^Department of Public Health, University of Copenhagen, Copenhagen, Denmark

**Keywords:** nanotechnology, regulatory pathways, GDPR, consent, pharmaceutical development, illicit drugs, GLP

This Research Topic, “*Reviews in Regulatory Science*”, features a number of publications from 2024 and 2025. These articles cover, among other topics, regulatory approaches to the GDPR and approaches to new technologies in pharmaceutical development.

Oku and Someya, both from The Japanese Pharmaceuticals and Medical Devices Agency (PMDA), analyzed trends in GLP-compliant non-clinical studies submitted to the PMDA from 2017 to 2023. The USA and Canada contributed the most GLP studies to the PMDA, followed by Japan, Europe, and the United Kingdom. The number of GLP studies from China and Taiwan has been on the rise over the last 3 years, reflecting China's growing development of new drugs, particularly in the oncology field. China is not a part of the OECD Mutual Acceptance of Data (MAD) framework, in which non-clinical safety studies conducted in test facilities that have been successfully inspected by a competent GLP-compliance monitoring authority in one OECD country are accepted by other OECD countries. The PMDA only accepts data from test facilities in non-MAD countries if the PMDA's product-based inspection of the studies conducted is successful.

The authors found a decrease in the percentage of studies conducted in Japan, which suggests a reduction in drug development activities in that country. They also found a considerable time lag for many of the studies, which were submitted to the PMDA later than to the United States or the European Union, and this is considered a serious issue for patients with life-threatening diseases in Japan.

Christofidou et al. made an important contribution to the discussion of the practical application of informed consent and how to interpret it in accordance with European legislation. The review investigated the gaps in the European General Data Protection Regulation (GDPR) regarding the interpretation and practical application of consent for the secondary use of health data. Furthermore the work discussed potential solutions. The GDPR's requirements for “informed consent” are not well-defined in contexts such as genome research. The review proposes using the Data Governance Act (“DGA”) and the concept of “data altruism” as a cohesive solution to this.

A systematic review by Chen et al. utilized bibliometric and visualization analyses of the core collection of Web of Science databases to evaluate the status and trends in the field of illicit drugs globally. The review included a total of 5,797 publications issued between 2015 and 2024. The literature on substance abuse research mainly focuses on addiction mechanisms, mental health impacts, and intervention strategies. Of interest is the rise in the clinical application of non-pharmacological approaches such as Mindfulness-Based Interventions and cognitive behavioral therapy.

Although the United States has made substantial contributions to the field of illicit drug research, they do not play a significant role in global research cooperation. With low levels of international cooperation, research remains domestically oriented, which may impede the global impact and innovative capacity of the research conducted. Data sharing, technological exchange, and collaborative actions among nations are instrumental in the establishment of a more efficient and coordinated global drug governance system. This system would better equip the international community to address the threats posed by drugs to public health and social security.

Agyralides discussed the impact of innovative technologies on the pharmaceutical development ecosystem. Technology is evolving rapidly, and it is being dominated by artificial Intelligence, including Machine Learning and the use of Big Data and Real-World Data (RWD) to produce Real-World Evidence (RWE). Nanotechnology is an interdisciplinary field that provides new opportunities for the manufacturing of devices and products with dimensions of a billionth of a meter. Artificial Neural Networks and Deep Learning mimic the human brain by combining computer science with new theoretical foundations for complex systems. The author also discussed technologies such as personalized medicines, gene therapy and CRISPR. The rapid development of new technologies significantly speeds up the process and reduces the costs for the development of new medicines, and offers more options for better, safer, and more effective treatments, with a more solid, data-driven and evidence-based approach for patients. However, a focus should be maintained on a safe and ethical data-sharing culture.

Nanotechnology was also the focus of the review by Rodríguez-Gómez et al. The integration of nanotechnology into healthcare led to the development of Nanotechnology-Enabled Health Products (NHPs), which show promise for making revolutionary advancements in medical treatments and diagnostics. NHPs have the potential to advance four key areas: nano-diagnosis, controlled drug delivery, treatment, and regenerative medicine. Despite their potential, navigating the regulations for these products remains complex. Rodríguez-Gómez et al. provided an excellent overview of the regulatory landscape for NHPs in the European Union and the United States, identifying the applicable requirements and the main regulatory guidelines currently available for meeting regulatory expectations.

The regulation of health technologies consistently lags behind rapid advancements in research and development, and the delay in establishing specific regulatory guidelines for NHPs is pronounced. The evolving regulatory landscape for NHPs across the EU and the US—and increasingly in emerging markets such as China and Japan—continues to face persistent hurdles. These include the absence of harmonized definitions, complex physicochemical characterization requirements, and the intricacies of evaluating nanotoxicity. Overall, efforts to modernize regulatory frameworks and encourage standardized testing, coupled with the emergence of AI-driven methodologies and the shift toward greener nanomanufacturing, signal a promising future for nanomedicine; however, further collaboration across scientific, governmental, and industrial spheres is essential to fully harness these opportunities.

